# Pilot mental workload analysis in the A320 traffic pattern based on HRV features

**DOI:** 10.3389/fnrgo.2025.1672492

**Published:** 2025-11-12

**Authors:** Jiajun Yuan, Bo Jia, Chenyang Zhang, Lu Tian, Han Yi, Lin Wei

**Affiliations:** 1 Flight Technology College, Civil Aviation Flight University of China, Guanghan, China; 2 China Eastern Airlines Technology Application Research and Development Center Co., Ltd., Shanghai, China; 3 School of Transportation and Logistics, Southwest Jiaotong University, Chengdu, China

**Keywords:** traffic pattern, mental workload, machine learning, HRV features, pilot

## Abstract

Pilot mental workload is a critical factor influencing flight safety, particularly during dynamic flight phases with high cognitive demands such as takeoff and landing. This study evaluates pilot workload across different flight phases (takeoff, climb, cruise, descent, and landing) using HRV (heart rate variability) features and machine learning methods. Heart rate data were collected through simulated A320 traffic pattern flight missions, combined with multidimensional task assessments, to obtain flight performance scores. Selected HRV features, Min_HR (minimum heart rate), SDNN (standard deviation of normal-to-normal intervals), SD2 (long-term variability index in Poincare Plot), Modified_csi (modified cardiac sympathetic index), were identified and used to train classifiers (RF, KNN, GBDT, XGBoost) for pilot mental workload level classification. The XGBoost model demonstrated optimal performance after feature selection, with accuracy increasing from 50.09% to 66.67% (a 16.58% improvement) and F1-score rising from 37.63% to 58.33% (a 20.70% improvement) compared with all HRV feature. The findings revealed selected HRV suppression during high-workload phases (landing) with the lowest performance scores, whereas HRV recovery and peak performance scores were observed in low-workload phases (cruise). This research establishes a reliable framework for real-time pilot mental workload monitoring and provides predictive insights into cognitive overload risks during critical flight operations.

## 1 Introduction

With the rapid advancement of aviation technology and the continuous implementation of novel aeronautical systems, human-machine interface systems in aircraft operations have grown increasingly complex, leading to a steady escalation in pilot mental workload (Wang P. et al., 2024). Statistical data indicate that approximately 70%~80% of civil aviation accidents and incidents are closely associated with human factors during flight (Kharoufah et al., 2018). Under high-intensity flight task loads, pilot frequently exhibit adverse physiological and psychological responses (Borghini et al., 2014) including cognitive latency, emotional irritability, operational distortion, and motor coordination impairment, all of which pose significant threats to aviation safety.

The A320 traffic pattern (a continuous task flow covering takeoff, climb, cruise, descent, and landing) differs from existing flight research that mostly focuses on single phases (Dussault et al., 2005). As a mainstream civil aircraft, the A320 has representative cockpit operation logic and task allocation, and analyzing workload in its traffic pattern is more in line with real flight scenarios where pilots continuously adapt to changing task loads, thus providing more practical guidance for improving civil aviation safety.

The inherently intangible concept of mental workload is typically assessed through multidimensional approaches encompassing subjective perception, flight performance, and physiological parameters (Young et al., 2014). Pilot provide self-reported ratings via scales or questionnaires to reflect subjective experiences (Mansikka et al., 2019). Performance-based evaluations, on the other hand, indirectly assess workload through analyses of operational proficiency, decision-making capability, and crisis response competence (Whitehurst, 2013) Physiological parameter assessments objectively infer workload through biological indicators, such as PPG (photoplethysmography) and EEG (electroencephalogram) signals (Young et al., 2014). The PPG signal detection demonstrates significant advantages in terms of stability, accessibility, and clinical relevance (Allen, 2007). A validation study of the Polar reported high agreement with ECG (Electrocardiogram) across activity intensities and wearing positions, with reduced motion artifacts when positioned on the arm (Coste et al., 2025). HRV features derived from PPG have shown significant correlations with task workload in VR flight simulations (Vallès-Català and Guerrero, 2025) and retained acceptable reliability under motion in field studies. An elevated workload is typically characterized as increased heart rate and a decrease HRV (Hebbar et al., 2021).

HRV refers to the variability of time intervals between consecutive heartbeats (NN intervals) and is a key physiological indicator reflecting the function of the autonomic nervous system (ANS). It quantifies fluctuations in heart rhythm to assess the balance between the sympathetic and parasympathetic nervous systems (Shaffer and Ginsberg, 2017). In this study, HRV data were extracted from PPG signals—PPG indirectly reflects the cardiac cycle by detecting changes in blood volume; after preprocessing, its signals can be converted into NN interval sequences, from which HRV features are further calculated (Schweizer and Gilgen-Ammann, 2025). This method has been validated as an effective alternative to traditional ECG chest straps in aviation scenarios. The ANS consists of the sympathetic and parasympathetic nervous systems, which collaboratively regulate cardiovascular activity: sympathetic activation increases heart rate and decreases HRV, while parasympathetic activation decreases heart rate and increases HRV (Billman, 2011, 2013). Common HRV features can be categorized into time-domain, frequency-domain, and non-linear indices (Malik et al., 1996). Among them, SDNN (a core time-domain index) has been confirmed to decrease with sympathetic activation during high-load phases such as takeoff and landing (Cao et al., 2019); SD2 (a long-term variability index in Poincare Plot) is positively correlated with pilots' autonomic regulatory capacity in continuous multi-phase tasks (Alaimo et al., 2022); Modified_csi (modified cardiac sympathetic index) quantifies sympathetic tone, and its decrease indicates excessive sympathetic activation, which is common in high-cognitive-load landing phases. Specifically, LF (0.04–0.15 Hz) is associated with sympathetic activity, and HF (0.15–0.4 Hz) with parasympathetic activity; an increase in LF/HF often indicates sympathetic dominance (e.g., high workload) (Meyer et al., 2019). A decrease in RMSSD reflects parasympathetic inhibition, which is associated with high load from complex decision-making tasks (Castaldo et al., 2015). A decrease in SD2 indicates impaired autonomic regulation, which is common in acute stress phases such as landing.

The traffic pattern plays a crucial role in pilot training by simulating a continuous multi-phase task flow that forces pilots to switch operational priorities (e.g., from thrust adjustment in the climb phase to altitude control in the descent phase), thereby enhancing situational awareness and emergency response capabilities. Additionally, significant differences in task complexity and time pressure exist across phases (e.g., landing requires simultaneous monitoring of glideslope and heading signals, while cruise only requires maintaining altitude and speed), providing a natural experimental setting for analyzing HRV changes under different workloads. During takeoff, a high workload has been shown to cause a decrease in SDNN (standard deviation of normal-to-normal intervals), which is significantly correlated with the airspeed error rate. During long-term cruises, reduced TP reflects fatigue accumulation (Soares et al., 2024). The high-precision operational demands during the approach reduce the RMSSD of the baseline values.

Existing HRV-based mental workload assessments have demonstrated efficacy in aviation, though most studies focus on single phases (e.g., cruise or emergencies); while some studies involve multi-phases (Alaimo et al., 2022), they do not systematically explore the impact of continuous workload changes across phases in the A320 traffic pattern on HRV features. The present study develops a multi-parameter assessment framework to capture pilot HRV dynamics and operational performance across traffic patterns. In this framework, standardized HRV features are combined with multidimensional flight performance indicators to construct a comprehensive feature vector, which serves as the input for a machine learning classifier to predict workload levels.

## 2 Materials and methods

### 2.1 Participants

A study was conducted in which 20 healthy Chinese male pilot cadets (aged between 21 and 26 years) from the Flight Technology College of the Civil Aviation Flight University of China participated in flight simulation missions. All participants had prior experience with the A320 aircraft, and the total simulated flight time they had completed (not the duration of this experiment) was 245 ± 32 h. The Edinburgh Handedness Inventory revealed that all participants were right-handed. They had normal or corrected-to-normal vision and hearing. The experiment was conducted between 9 a.m. and 5 p.m. Prior to the commencement of the experiment, the use of any drug, alcohol, or caffeine was prohibited. Prior to participation, all subjects provided written informed consent, and financial compensation was provided for their time. The study was conducted in accordance with the principles of the Declaration of Helsinki and received approval from the Ethical Review Board of Civil Aviation Flight University of China (No. 2024-7).

### 2.2 Experimental equipment

**Figure 1:**
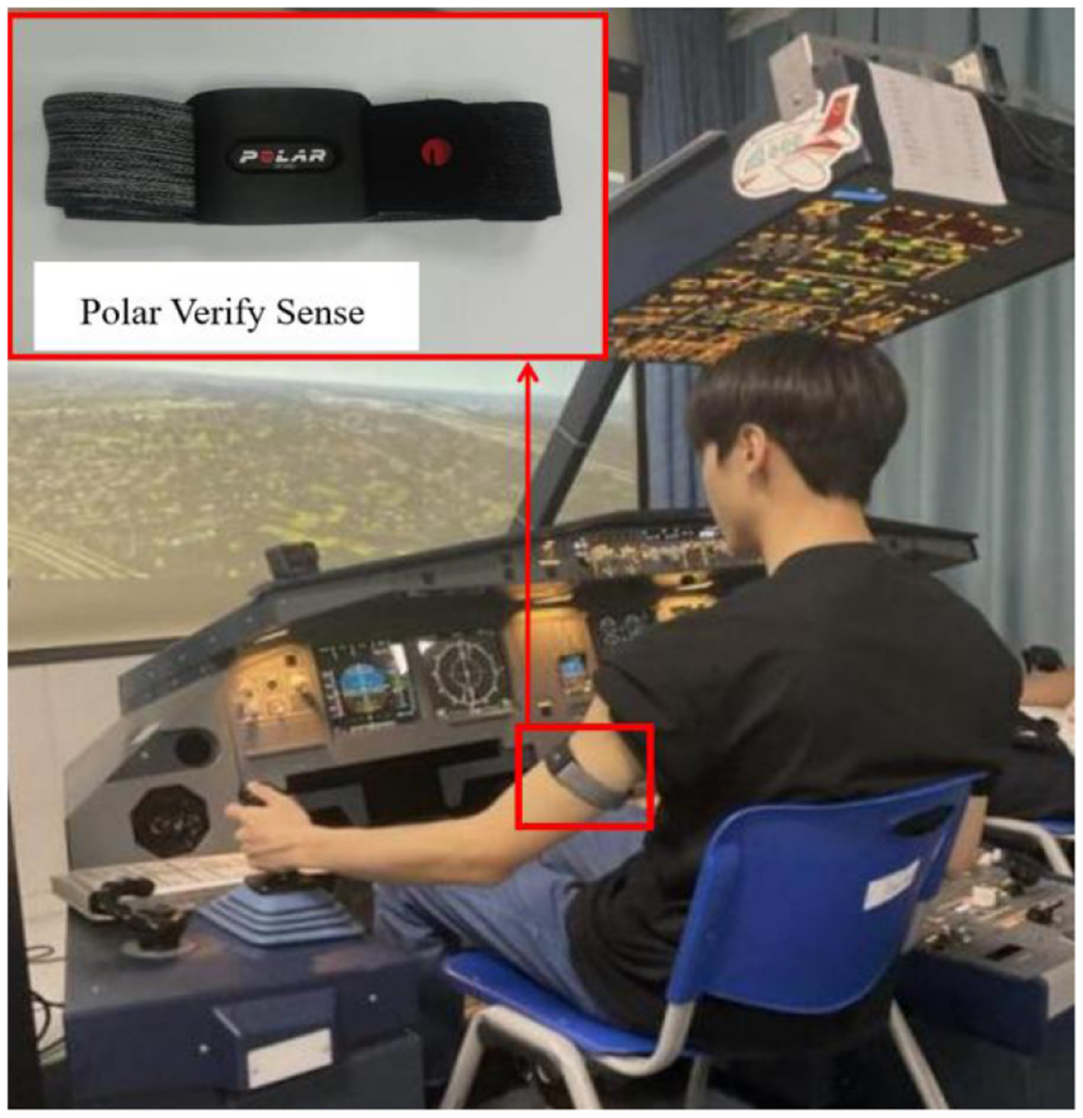
Flight simulator and polar verity sense sensor.

The simulator employed in the experiments was an A320 flight training device (FTD; Aviation Flight Simulation Research Centre Co., Ltd.), as illustrated in Figure 1. The A320 FTD is a high-quality and cost-effective simulation hardware equipment solution, with a 1:1 scale simulation of each functional component. Pilot HRV was monitored using the Polar Verity Sense, a device that enables contact-free HRV monitoring, offering a robust alternative to ECG chest straps, as illustrated in Figure 1.

### 2.3 Experimental procedures

Before experiment, participants are required to sign an informed consent form and complete a personal information form to collect their basic information and flight experience. Furthermore, a 15-min familiarization period with the FTD was allocated, during which the subjects were fitted with a Polar Verity Sense. This was followed by a 5-min rest period.

**Figure 2:**
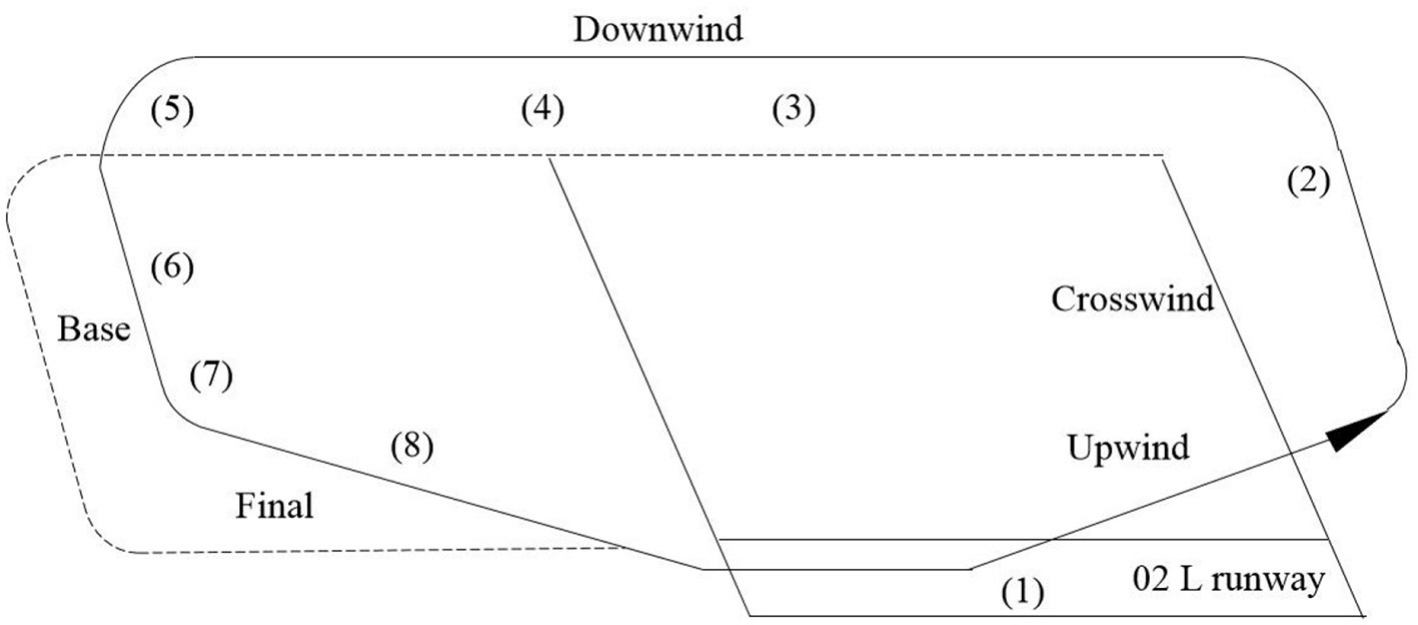
Flight procedure of A320 traffic pattern.

The formal experiment is described as follows: The selected traffic pattern comprised runway 02 L at Chengdu Shuangliu International Airport, and the additional information comprised A320, lightweight, calm or light wind, ceiling and visibility okay, visibility greater than 10 km, dry runway, and no autopilot throughout the experiment. The flight procedure (illustrated in Figure 2) for the subjects is as follows:

1) The subjects were required to take off from the Shuangliu 02 L runway at a heading of 024°, with a target altitude of 4,900 ft.
2) Subsequent to climbing to 2,000 ft, a left turn was to be executed at a bank of 30° to a downwind heading of 204°.
3) The downwind leg was intercepted, the altitude was maintained at 4,900 ft, with a track of 204°, and flaps were set to position 1 at a suitable speed.
4) The initiation of timing was initiated at 45 seconds when aligned with the runway entrance, and flaps were set to position 2 at 35 seconds into the timing.
5) Third turn was initiated after 45 seconds, with the descent rate being controlled at 400 feet per minute.
6) The aircraft should be aligned with the base leg, the base heading should be set to 294°, the landing gear should be set to the down position, the flap should be set to position 3, and then the flap should be set to full.
7) A base turn should be executed when the localizer signal is one dot, and a turn toward the final approach should be made with a 30° bank.
8) The aircraft's position on the normal glidepath should be verified through reference to the glideslope and flight director, with the objective of ensuring the successful execution of the landing.

Concurrently, the evaluator documented the instances at which the participant successfully completed each flight phase, employing a flight performance scale (see Section 2.4) to record their performance.

Takeoff requires precise throttle adjustment to avoid exceeding engine parameters, while monitoring the aircraft's movement trends and takeoff decision conditions, resulting in high workload (Zhang et al., 2019); landing requires simultaneous adjustment of the descent rate, monitoring of glideslope and heading signals, and preparation for go-around, making it the phase with the highest information processing demand and workload. This stage demands meticulous monitoring of numerous parameters, ensuring both the safe climb of the aircraft and the clearance of any obstacles. This is a particularly arduous task for the pilot. It is during this phase that the highest levels of workload are experienced by the pilot.

During the climb phase, the pilot must increase thrust to prevent engine damage from prolonged high-power operation and complete configuration retraction. The pilot must also judge the timing for the aircraft to level off and ensure that it does not exceed a pre-selected altitude. The number of parameters requiring focus is reduced, and the amount of manipulation is decreased, resulting in a lower workload.

The pilot's workload is minimal during the cruise phase, with only the need to maintain the aircraft's altitude and green-dot speed and to time the deployment of flaps after passing the landing area. The aircraft's state is stable, and the number of parameters to be monitored is minimal. This phase is characterized by minimal effort expended by the pilot and minimal workload perception.

The descent phase entails the pilot manipulating the aircraft and adjusting the throttle to descend at an appropriate rate. It is required to focus on the aircraft's status parameters, such as heading, speed, altitude, and N1 RPM, as well as the height-to-distance ratio, instruments to determine the timing for turns, and the establishment of the landing configuration. It is imperative that the pilot ensures the aircraft is on the correct descent path and descending along a normal gradient. In comparison to the takeoff and landing phases, the amount of manipulation is reduced; however, continuous attention to aircraft parameters is imperative. The workload is moderately reduced.

The landing phase is characterized by the necessity for the pilot to regulate the aircraft's descent at the optimal rate, whilst concurrently maintaining vigilant oversight of the aircraft's height-to-distance ratio, and effecting requisite adjustments. They must also observe the heading and glideslope signals, adjust the flight path as required, listen to the tower frequency, and prepare for a possible go-around at any time. The landing phase necessitates the management of the aircraft's landing while concurrently considering the relevant flight parameters and the aircraft's actual trajectory. This phase necessitates the most extensive information processing and effort from the pilot, consequently rendering it the most demanding in terms of workload.

### 2.4 Flight performance scale

This experiment focused on the dimensions of flight capabilities that pilots should possess, as outlined in the literature. It incorporates the opinions of senior flight instructors from airline companies and flight training units and refers to the pilot training syllabus. The indicators for evaluating the flight performance were determined based on the features of the simulated flight experiment, as shown in Table 1. The evaluation of the programme's performance was conducted using a 100-point scale, ranging from 0 to 100 points. Scoring was conducted by a flight evaluator with rich experience and impartial attitude following the completion of each phase of the flight phase, and higher the score means better the flight performance.

**Table 1:** Flight performance scale.

**Program Phase**	**Flight control**	**Navigation**	**Communication**	**Decision**	**Total**
Take off					
Climb					
Cruise					
Descent					
Landing					

The choice of flight performance scores as a workload assessment indicator is based on the following reasons: (1) In aviation scenarios, pilot workload is ultimately reflected in operational performance; flight performance scores (e.g., flight control accuracy, navigation precision) directly reflect the impact of workload on task execution, whereas subjective scales like NASA-TLX are susceptible to individual subjective biases (e.g., differences in workload tolerance). (2) Existing studies have shown that in simulated flight tasks, the correlation between flight performance scores and HRV indices is significantly higher than that between subjective scales and HRV, verifying the indicator's effectiveness. (3) The flight performance scale in this study integrates the experience of senior airline instructors and training syllabi, covering multiple dimensions of flight control, navigation, communication, and decision-making (Li et al., 2018), enabling comprehensive capture of performance differences across different workload phases.

## 3 Data analysis

### 3.1 Analysis of the flight performance scale

An analysis of participants' flight performance scores during traffic pattern revealed that the characteristics of flight phases, such as mental workload intensity, operational complexity, and time pressure, significantly impact pilot performance across a range of dimensions, including flight control, navigation, communication, and decision-making (see Table 2 for details). Table 2 presents the mean ± standard deviation of flight performance scores for the participants across each flight phase. Pearson correlation analysis was used to verify the correlation between workload and performance, revealing a significant negative correlation (r = −0.73, *p* < 0.01) between workload (comprehensively assessed based on HRV features) and total performance scores, confirming that high workload significantly impairs operational performance. This finding serves to validate the hypothesis that an elevated mental workload has a detrimental effect on operational effectiveness.

**Table 2:** Scores of flight performance scale.

**Program phase**	**Flight control**	**Navigation**	**Communication**	**Decision-making**	**Average**
Take off	84.1 ± 10.2	88.9 ± 7.9	85.2 ± 8.6	85.5 ± 6.4	85.4 ± 8.2
Climb	88.7 ± 9.8	91.2 ± 9.6	89.1 ± 7.2	91.8 ± 6.2	90.5 ± 7.6
Cruise	92.5 ± 9.4	93.4 ± 10.2	93.8 ± 6.5	95.2 ± 4.2	92.9 ± 8.4
Descent	86.9 ± 9.7	89.1 ± 8.5	87.3 ± 8.3	88.4 ± 8.0	87.9 ± 7.3
Landing	81.2 ± 10.4	88.6 ± 7.2	83.1 ± 9.1	82.7 ± 9.1	82.9 ± 6.9

Operational complexity exerts a predominant influence on flight control scores, with phases characterized by higher dynamic operational demands (e.g., takeoff and landing) demonstrating reduced flight control performance. Standardization of tasks has been shown to enhance navigation and communication efficiency (e.g., climb and cruise phases) by mitigating human error risks, resulting in higher scores. The correlation between decision-making pressure and cognitive resource allocation is also demonstrated; multitasking scenarios in high-workload phases result in degraded decision quality, whereas low-workload phases demonstrate superior decision-making performance.

### 3.2 Pre-processing of PPI data

Preprocessing raw PPI (pulse-to-pulse interval) data acquired from POLAR sensors is critical for ensuring the accuracy of the physiological analysis. The workflow commences with the cleansing of the data via the implementation of threshold-based filtering and the detection of statistical outliers, with the objective of the elimination of physiologically implausible data points. The temporal continuity of the signal is then ensured by correcting transient signal losses or artifacts using linear interpolation (Benchekroun et al., 2022). The presence of high-frequency noise, attributable to either motion artifacts or sensor instability, is addressed through the implementation of moving average filters or Butterworth low-pass filters. The non-uniform PPI time series was resampled at fixed frequencies to generate uniformly spaced signals for the purpose of standardized HRV analysis. Data segments contaminated by intense physical activity were further excluded based on synchronized accelerometer thresholds or user-annotated activity logs. All preprocessing steps were implemented using Python open-source libraries, specifically including pandas (for data cleaning and management), numpy (for numerical computation and interpolation), scipy.signal (for Butterworth low-pass filtering), and hrv-analysis (for PPI data conversion) (Champseix et al., 2021), ensuring reproducibility.

### 3.3 HRV features extraction and selection

After preprocessing the PPI data, HRV features were extracted using the Hrv-analysis package in the Python environment. A time window of 30 s was selected, with a 40% overlap between the adjacent time windows. After processing, a total of 30 HRV features were extracted, including SDNN, Mean_NNI, STD_HR, Min_HR, and LF/HF, as shown in Table 3.

**Table 3:** HRV features.

**Features**	**Definition**	**Features**	**Definition**
Mean_NNI	Mean value of heartbeat intervals	Std_hr	Standard deviation of heart rate
SDNN	Standard deviation of NN intervals	LF	Low frequency power
SDSD	Standard deviation between adjacent NN intervals	HF	High frequency power
PNNI_20	NN interval greater than 20 milliseconds per cent	LF/HF	Ratio of low frequency power to high frequency power
PNNI_50	Percentage of NN intervals greater than 20 milliseconds	LFNU	Normalized low frequency power
NNI_20	Number of times adjacent NN intervals differ by more than 20 milliseconds	HFNU	Normalized high frequency power
NNI_50	Number of times adjacent NN intervals differ by more than 50 milliseconds	TP	Total energy of the spectrum
RMSSD	Root mean square difference between adjacent NN intervals	VLF	Energy of the power spectrum
Median_NNI	Median of NN intervals	SD1	Standard Deviation 1 in Poincare Plot
Range_NNI	Range of NN intervals	SD2	Standard deviation 2 in Poincare Plot
CVSD	Coefficient of variation for continuous differences	SD2/SD1	Ratio of standard deviation 2 to standard deviation 1
CVNNI	Coefficient of variation of NN intervals	CSI	Cardiac sympathetic index
Mean_HR	Mean heart rate	CVI	Cardiac vagal index
Max_HR	Maximum heart rate	Modified_csi	Modified cardiac sympathetic index
Min_HR	Minimum heart rate	^Triangular_index^	Trigonometric index

Due to the limited number of participants (less than 50) available for consideration, following the implementation of the Shapiro-Wilk test (Razali and Wah, 2011), it was determined that these features did not adhere to a normal distribution. The Kruskal-Wallis test only determines whether features exhibit statistical differences across flight phases (*p* < 0.05), but cannot evaluate their contribution to workload classification tasks. Consequently, the random forest algorithm employed to rank the importance of these HRV features. The specific implementation parameters of the RF feature importance ranking are as follows: number of decision trees (n_estimators) = 100, maximum tree depth (max_depth) = 5, feature sampling method (max_features) = “sqrt,” random seed (random_state) = 42; “node impurity reduction” (Gini coefficient reduction) was used as the metric for feature importance. After ranking the importance of 30 HRV features, the top 5 features that also passed the Kruskal-Wallis test (*p* < 0.05) were selected, ultimately identifying Min_HR, SDNN, SD2, and Modified_csi as key features, as see in Table 4.

**Table 4:** Importance and significance of HRV features.

**Features**	**Significance**	**Importance**	**Features**	**Significance**	**Importance**
Mean_NNI	0.080	0.04064	Std_hr	0.075	0.04197
**SDNN**	**0.044**	**0.03369**	LF	0.178	0.02978
SDSD	0.117	0.02922	HF	0.397	0.03598
PNNI_20	0.655	0.02645	LF_HF_ratio	0.151	0.02946
PNNI_50	0.067	0.01789	LFNU	0.151	0.02934
NNI_20	0.779	0.02591	HFNU	0.151	0.02942
NNI_50	0.083	0.01797	Total_power	0.286	0.03157
RMSSD	0.113	0.02943	VLF	0.152	0.03179
Median_NNI	0.091	0.05242	SD1	0.116	0.02944
Range_NNI	0.056	0.03614	**SD2**	**0.046**	**0.03467**
CVSD	0.116	0.03949	Ratio_SD2_SD1	0.111	0.02903
CVNNI	0.055	0.03618	CSI	0.111	0.02853
Mean_HR	0.163	0.04406	CVI	0.070	0.03738
Max_HR	0.244	0.05581	**Modified_csi**	**0.026**	**0.03250**
**Min_HR**	**0.017**	**0.04573**	^Triangular_index^	0.198	0.01798

Bold values indicate HRV features selected by Kruskal-Wallis test (*p* < 0.05) and random forest feature importance ranking, which are used as key features for subsequent workload classification.

The significance threshold was set at p < 0.05, and it was found that only Min_HR, SD2, SDNN, and Modified_csi met this threshold. The reason only a limited number of HRV features showed significant differences may be: (1) Other HRV features are susceptible to transient interference during phase transitions, leading to increased variability and reduced statistical significance; (2) The traffic pattern in this study is characterized by continuous dynamic workload changes, and indices like Min_HR and SDNN better reflect long-term (30s time window) autonomic regulation trends, making them more compatible with the workload changes of continuous tasks. Thus, these four were selected as the chosen indicators to reflect the intensity differences across different flight phases.

### 3.4 Machine learning algorithms

#### 3.4.1 Random forest

RF is an ensemble learning method that is based on bagging (bootstrap aggregation) (Breiman, 2001). It constructs multiple decision trees and combines their predictions (majority voting for classification tasks and averaging for regression tasks) to improve the generalization ability. Its core principles include sample randomness (each tree is trained using bootstrap sampling) and feature randomness (each tree randomly selects a subset of features during the splitting). RF is suitable for high-dimensional data, robust to noise, but relatively less interpretable, making it ideal for multi-stage workload classification tasks using pilot HRV features. The prediction formula for RF is:


$$\begin{array}{l} \overset{ˆ}{y}=\text{mode}\left(\left\{T_{1}\left(\text{x}\right),T_{2}\left(\text{x}\right),\ldots ,T_{B}\left(\text{x}\right)\right\}\right) \end{array}$$ (1)

In the context of pilot workload classification, $\overset{ˆ}{y}$ denotes the predicted class label (workload level), **x** represents the input HRV feature vector (e.g., SDNN, LF/HF, RMSSD), *T*_*B*_(**x**) is the prediction of the *b*-th tree in the ensemble, *B* is the total number of trees.

#### 3.4.2 K-nearest neighbors

KNN is a non-parametric lazy learning algorithm that performs classification or regression by measuring the similarity between samples (Halder et al., 2024). The core assumption is that adjacent samples in the feature space share similar physiological response patterns. KNN is suitable for small-scale incremental datasets; however, high-dimensional data (e.g., HRV features) may suffer from the curse of dimensionality, requiring PCA (principal component analysis)-based dimensionality reduction to enhance robustness. Owing to its high computational complexity and poor real-time performance, KNN is best suited for the short-term dynamic analysis of pilot workloads, such as detecting transient stress states during critical flight phases.


$$\begin{array}{l} \overset{ˆ}{y}=\text{mode}\left(\left\{y_{1},y_{2},\ldots ,y_{K}\right\}\right) \end{array}$$ (2)

In the equation, *y*_*i*_ denotes the workload label of the *i*-th neighbor of sample *x*.

#### 3.4.3 Gradient boosting decision tree

Gradient boosting decision tree (GBDT) (Friedman, 2001) incrementally constructs decision trees to iteratively correct residuals (errors) from prior models through additive modeling, a linear combination of weak learners (decision trees), and gradient descent, which optimizes the loss function by following the negative gradient direction. The GBDT excels at capturing cross-phase cumulative workload effects in flight missions, such as prolonged stress during flight tasks, but its slower computational speed and susceptibility to overfitting necessitate careful tuning of the learning rate (shrinkage) to control update step sizes and ensure robust generalization.


$$\begin{array}{ll} p_{i}= & \frac{1}{1+e^{-{\overset{ˆ}{y}}_{i}}},{\overset{ˆ}{y}}_{i}=\overset{T}{\underset{t=1}{\sum }}f_{t}\left(x_{i}\right) \end{array}$$ (3)

$$\begin{array}{l} l\left(y_{i},{\overset{ˆ}{y}}_{i}\right)=-y_{i}\log\left(p_{i}\right)-\left(1-y_{i}\right)\log\left(1-p_{i}\right) \end{array}$$ (4)

$$\begin{array}{l} r_{i}^{\left(t\right)}=y_{i}-p_{i}^{\left(t-1\right)} \end{array}$$ (5)

In the equation, *p*_*i*_ denotes the predicted probability for the i-th sample, ${\overset{ˆ}{y}}_{i}$ represents the linear prediction value of the *i*-th sample. *T* is the total number of decision trees, and *f*_*t*_(*x*_*i*_) stands for the prediction output of the *t*-th decision tree for the *i*-th sample *x*_*i*_. $l\left(y_{i},{\overset{ˆ}{y}}_{i}\right)$ signifies the loss function for the *i*-th sample, with *y*_*i*_ being the true label of the *i*-th sample. $r_{i}^{\left(t\right)}$ indicates the residual of the *i*-th sample at the *t*-th iteration, and $p_{i}^{\left(t-1\right)}$ refers to the predicted probability of the *i*-th sample after the (*t*−1)-th iteration.

#### 3.4.4 XGBoost(Extreme Gradient Boosting)

XGBoost, an optimized implementation of the GBDT, significantly enhances generalization and computational efficiency by incorporating regularization terms (*L*_1_/*L*_2_ penalties) to control model complexity and leveraging the second-order Taylor expansion to refine the objective function (Wang D. et al., 2024). Key advancements include second-derivative optimization, which accelerates convergence by utilizing both first- and second-order gradients of the loss function, and parallelized feature presorting to expedite split-point selection during tree construction. These innovations make XGBoost particularly effective for high-precision dynamic workload prediction, such as the real-time monitoring of pilot HRV features to detect abrupt workload shifts during critical flight phases (e.g., turbulence recovery or emergency maneuvers).


$$\begin{array}{l} L=\overset{n}{\underset{i=1}{\sum }}\overset{C}{\underset{c=1}{\sum }}y_{i,c}\log\left(p_{i,c}\right)+\overset{T}{\underset{t=1}{\sum }}\Omega \left(f_{t}\right) \end{array}$$ (6)

In the equation, $p_{i,c}=\frac{e^{{{\overset{ˆ}{y}}_{i}}_{,c}}}{\overset{C}{\underset{c=1}{\sum }}e^{{{\overset{ˆ}{y}}_{i}}_{,c}}}$ represents the class probability, where *C* denotes the number of classes (5 levels in this study: high, relatively high, medium, relatively low, and low). The predicted value of a leaf node is given by


$$\begin{array}{l} w_{t,c}^{*}=-\frac{\sum_{i\in I_{t}}g_{i,c}}{\sum_{i\in I_{t}}h_{i,c}+\lambda } \end{array}$$ (7)

Here, $g_{i,c}=\frac{\partial l}{\partial \theta_{i,c}},h_{i,c}=\frac{\partial^{2}l}{\partial {{\overset{ˆ}{y}}_{i}}_{,c}^{2}}$, increasing λ (regularization coefficient) helps prevent overfitting on small-sample pilot data.

### 3.5 Results

Feature selection was conducted in accordance with two approaches: (1) Full feature input: using all 30 preprocessed HRV features (not a feature selection process, only used as a baseline for comparison); (2) Selected feature input: using 4 key HRV features (Min_HR, SDNN, SD2, Modified_csi) selected via Kruskal-Wallis test (*p* < 0.05) and RF feature importance ranking. The feature data underwent label encoding and robust standardization (based on the median and interquartile range) to enhance robustness against outliers. The dataset was split into a 90% training set and 10% test set using stratified sampling to maintain the class distribution. This split ratio was chosen because: the total number of samples (1,200 HRV feature samples across phases) was small; a 90% training set ensured sufficient data for model learning, while a 10% test set evaluated generalization without overfitting. In order to address issues of dimensionality and class imbalance, the input data underwent a reshaping process for model compatibility, with categorical labels being converted to discrete workload levels and SMOTE (synthetic minority over-sampling technique) oversampling being applied to balance minority classes. The models were trained and evaluated via five-fold cross-validation, with mean accuracy, precision, recall, Cohen's Kappa, and F1-score calculated as performance metrics, as detailed in Table 5. This study compared the performance differences of four machine-learning classifiers (RF, KNN, XGBoost, and GBDT) when using all HRV features vs. filtered statistically selected HRV features.

**Table 5:** Comparison of classifier results.

**Classifier**	**All HRV features**				**Selected HRV features**			
**Performance**	**RF**	**KNN**	**XGBoost**	**GBDT**	**RF**	**KNN**	**XGBoost**	**GBDT**
Accuracy	0.4829	0.5658	0.5009	0.4883	0.5874	0.5964	0.6667	0.6216
Precision	0.4737	0.5568	0.4984	0.4857	0.5770	0.5787	0.6587	0.6177
Recall	0.4813	0.5713	0.5091	0.4924	0.5802	0.5974	0.6695	0.6259
Cohen's Kappa	0.4829	0.5658	0.5009	0.4883	0.5874	0.5964	0.6667	0.6216
F1 Score	0.3536	0.4572	0.3763	0.3606	0.4843	0.4956	0.5833	0.5270

The findings indicate that feature selection significantly enhances model performance, with all classifiers demonstrating marked improvements in metrics (Accuracy, Precision, Recall, F1, and Cohen's Kappa) after adopting selected HRV features. The XGBoost model demonstrated the most substantial enhancement in performance, with accuracy increasing from 50.09% to 66.67% (+16.58%), and the F1-score improving from 37.63% to 58.33% (+20.70%). A subsequent analysis of the selected features revealed that the model performance was ranked as XGBoost > GBDT > KNN > RF. The findings demonstrate that XGBoost attained optimal values for accuracy (66.67%), recall (66.95%), and F1-score (58.33%), thereby evidencing its superior robustness in the context of high-dimensional, small-sample HRV data analysis. The Cohen's Kappa coefficient approached moderate agreement, significantly outperforming other models, indicating an enhanced capability to mitigate misclassification risks (e.g., categorizing high workload as moderate).

The feature selection process was undertaken to optimize the model input space by eliminating redundant noise (e.g., time-domain statistic SDNN), while retaining autonomic nervous system-related features. This dimensionality reduction has been shown to alleviate the so-called “curse of dimensionality,” particularly with regard to the improvement of generalization in KNN (+3.06% accuracy) and XGBoost (+16.58% accuracy). The superior performance of XGBoost is attributed to its regularization mechanisms (L1/L2 penalties) and second-order derivative optimization (Hessian matrix), which effectively suppress overfitting and accelerate convergence (Fernández-Morales et al., 2022). Furthermore, its tree-based feature interaction modeling has been shown to better capture non-linear relationships between HRV metrics and workload levels in comparison to other classifiers.

Table 6 presents the mean values of the HRV features across distinct flight phases, along with the average flight performance ratings. These metrics reflect the dynamic associations between ANS activity and pilot workload. Min_HR was highest during landing and lowest during cruise. This is due to the fact that workload (both physical and cognitive) activates the sympathetic nervous system, elevating overall heart rates and sustaining Min_HR above baseline levels due to persistent sympathetic activation. The lower Min_HR during cruise indicates parasympathetic dominance (resting state). SD2 (long-term variability in Poincaré plots) exhibited a peak during cruise and a decline to its lowest level during landing, indicating stronger long-term autonomic regulation during cruise and diminished adaptive capacity under acute stress during landing. Concurrently, SDNN (overall HRV) exhibited a similar trend, peaking during cruise and reaching its nadir during landing, thereby aligning with the reduced global HRV observed under conditions of elevated workload, attributable to vagal tone inhibition and sympathetic activation (Gilboa et al., 2008).

**Table 6:** Average values of selected HRV features in different flight phases.

**Featuress**	**Takeoff**	**Climb**	**Cruise**	**Descent**	**Landing**
Min_HR (bpm)	76.85	59.98	41.86	71.88	78.75
SD2 (ms)	37.57	117.01	357.82	54.52	26.51
SDNN (ms)	29.54	89.09	263.42	43.12	21.49
Modified_csi	333.08	1,365.78	5,595.56	489.07	212.40
Average scores	85.4 ± 8.2	90.5 ± 7.6	92.9 ± 8.4	87.9 ± 7.3	82.9 ± 6.9

Combined with the HRV features and flight performance scores in Table 6, the workload differences across phases are further verified: the takeoff phase has a relatively high Min_HR (76.85 bpm) and low SDNN (29.54 ms), corresponding to an average flight performance score of 85.4, reflecting high workload; the landing phase has the highest Min_HR (78.75 bpm) and lowest SDNN (21.49 ms), with the lowest performance score (82.9), confirming it as the highest workload phase—consistent with existing research that landing requires the most information processing.

Modified_csi was the highest during cruise and the lowest during landing. Modified_csi values during cruise may reflect residual sympathetic activity from prior high-stress phases (e.g., takeoff). A low workload during cruise promotes parasympathetic dominance (high SDNN/SD2), facilitating physiological recovery. Conversely, the sympathetic surge during landing (high Min_HR, low SDNN/SD2) correlates with cognitive overload and degrades the decision-making accuracy.

## 4 Discussions

This study examined the relationship between heart rate variability (HRV) features and pilot workload across the A320 traffic pattern. The findings confirm HRV as a sensitive workload indicator. During high-workload landing phases, Min_HR increased while SDNN and SD2 decreased, reflecting sympathetic activation and parasympathetic withdrawal (Tulppo et al., 2005). Conversely, during low-workload cruise, Min_HR decreased and SDNN/SD2 increased, indicating stronger autonomic regulation. Moreover, Modified_csi peaked in cruise and dropped in landing, further verifying the suppressive effect of workload on sympathetic tone regulation (Jeppesen et al., 2014).

The XGBoost model achieved 66.67% accuracy, which is lower than results reported in studies focusing on isolated phases (Wilson, 2002). Three factors likely contributed: (1) the small sample (20 cadets) limited training data, particularly for high-workload samples, and residual class imbalance persisted despite SMOTE (Chawla et al., 2002); (2) workload transitions across the traffic pattern are continuous, complicating classification compared with binary contrasts (e.g., emergency vs. cruise); (3) HRV features are sensitive to individual baseline differences, and the absence of individualized correction may have reduced feature discriminability.

Despite these limitations, this study contributes in two important ways. First, it systematically examined dynamic HRV changes across the entire traffic pattern, offering a more comprehensive understanding of workload fluctuations in realistic operations. Second, it introduced Modified_csi, rarely applied in aviation studies, which demonstrated discriminative potential for workload assessment.

Three main limitations must be acknowledged. The small and homogeneous sample (male cadets) restricts generalizability; future studies should include larger, more diverse groups to validate findings. The simulator-based setting, while controlled, lacks ecological stressors such as turbulence and ATC demands; in-flight studies are necessary for external validity. Finally, the reliance on HRV alone may be insufficient; multi-modal data (e.g., EEG, eye tracking) should be integrated to improve model accuracy (Jap et al., 2009).

Building on these findings, this study proposes a real-time workload monitoring framework. Integrated into cockpit warning systems, wearable HRV devices can continuously extract features and feed them into the model, triggering alerts when sustained high workload is detected. Integration into simulator training could also provide cadets with real-time workload feedback, helping them adjust operation rhythm and enhance training efficiency.

## 5 Conclusions

A systematic assessment of the pilot workload across different flight phases (takeoff, climb, cruise, descent, and landing) was conducted using a simulated A320 traffic-pattern flight experiment. This assessment integrates HRV features, machine learning methods, and flight performance scores. Thirty HRV features were extracted from the raw PPI data, and four key features (Min_HR, SD2, SDNN, and Modified_csi) were identified using the Kruskal-Wallis test and random forest algorithm to reflect intensity differences across flight phases. Four machine learning classifiers—RF, KNN, GBDT, and XGBoost—were employed to predict workload levels based on all HRV features and selected key features, and their performances were compared. Major conclusions:

(1) During high-workload phases (e.g., landing), HRV was significantly suppressed, resulting in the lowest performance scores. In contrast, HRV recovery and peak performance scores were observed during low-workload phases (e.g., cruises). This indicates that HRV features effectively reflect changes in the pilot workload.

(2) The XGBoost model demonstrated the best performance after feature selection, with an accuracy improvement of 16.58% and an F1-score improvement of 20.70% compared with using all features. This highlights XGBoost's superior robustness in handling high-dimensional, small-sample HRV data, and its ability to reduce misclassification risks.

(3) The workload intensity, operational complexity, and time pressure of different flight phases significantly impact the pilot performance in terms of flight control, navigation, communication, and decision-making. A negative correlation exists between high workload and overall performance, confirming the hypothesis that high workload reduces operational efficiency.

## Data Availability

The raw data supporting the conclusions of this article will be made available by the authors, without undue reservation.
